# Efficient and Stable Routing Algorithm Based on User Mobility and Node Density in Urban Vehicular Network

**DOI:** 10.1371/journal.pone.0165966

**Published:** 2016-11-17

**Authors:** Yusor Rafid Bahar Al-Mayouf, Mahamod Ismail, Nor Fadzilah Abdullah, Ainuddin Wahid Abdul Wahab, Omar Adil Mahdi, Suleman Khan, Kim-Kwang Raymond Choo

**Affiliations:** 1 Department of Electrical, Electronic and Systems Engineering, Faculty of Engineering and Built Environment, Universiti Kebangsaan Malaysia, Bangi, Selangor, Malaysia; 2 Department of Computer System & Technology, Faculty of Computer Science & Information Technology Building, University of Malaya, Kuala Lumpur, Malaysia; 3 Department of Information Systems and Cyber Security, University of Texas, San Antonio, Texas, United States of America; 4 Information Assurance Research Group, Advanced Computing Research Centre, University of South Australia, Adelaide, Australia; Beihang University, CHINA

## Abstract

Vehicular ad hoc networks (VANETs) are considered an emerging technology in the industrial and educational fields. This technology is essential in the deployment of the intelligent transportation system, which is targeted to improve safety and efficiency of traffic. The implementation of VANETs can be effectively executed by transmitting data among vehicles with the use of multiple hops. However, the intrinsic characteristics of VANETs, such as its dynamic network topology and intermittent connectivity, limit data delivery. One particular challenge of this network is the possibility that the contributing node may only remain in the network for a limited time. Hence, to prevent data loss from that node, the information must reach the destination node via multi-hop routing techniques. An appropriate, efficient, and stable routing algorithm must be developed for various VANET applications to address the issues of dynamic topology and intermittent connectivity. Therefore, this paper proposes a novel routing algorithm called efficient and stable routing algorithm based on user mobility and node density (ESRA-MD). The proposed algorithm can adapt to significant changes that may occur in the urban vehicular environment. This algorithm works by selecting an optimal route on the basis of hop count and link duration for delivering data from source to destination, thereby satisfying various quality of service considerations. The validity of the proposed algorithm is investigated by its comparison with ARP-QD protocol, which works on the mechanism of optimal route finding in VANETs in urban environments. Simulation results reveal that the proposed ESRA-MD algorithm shows remarkable improvement in terms of delivery ratio, delivery delay, and communication overhead.

## Introduction

Drivers must be timely informed about perilous traffic situations, such as accidents, extreme weather, and low road traction, to experience safe and relaxed driving. The intelligent transportation system (ITS) is composed of vehicles and road side units (RSUs), which act and communicate as wireless nodes. The technology of wireless access in vehicular environments (WAVE) is a state-of-the-art technique used for ITS. This technology facilitates the vehicles to establish wireless communication and consequently form a vehicular ad hoc network (VANET) [1]. The modes of communication in VANETs include vehicle-to-vehicle (V2V) and vehicle-to-infrastructure (V2I) modes[2, 3].Given these communication modes, VANETs can be used in various applications and help provide important data to drivers and pedestrians. VANET applications can be categorized into two. One is safety application(such as collision avoidance at the intersection, public safety, sign extension, and vehicle diagnostics and other vehicle information), which enhances road safety and eliminates accidents [4–6].The other is non-safety application[7, 8], which focuses on the comfort of drivers and passengers and improving traffic efficacy. The traditional wireless communication technologies are not readily adaptable for vehicular communications. The inherent characteristics of VANETs, such as its frequent disconnections and high mobility of the vehicles, cause unstable communication link among vehicles [9–12]. Using RSUs is an option to solve this issue and facilitate communication among vehicles. However, the deployment of RSUs is a costly solution. Hence, relying only on the V2V communication mode is the most critical issue in VANETs when the deployment of RSUs is impractical. However, the frequent changes in topology and intermittent connectivity must be considered in advanced in designing an efficient and stable V2V routing protocol. In [13–15], various routing protocols for urban VANETs have been proposed. However, these protocols cannot meet the various quality of service (QoS) metrics required by diverse applications. Moreover, the objectives of the most current routing protocols focus on either the most efficient route with the minimum hop count or the most stable route with the longest link duration. The balance between an efficient route (minimum hop count) and a stable route (longest link duration) in designing an optimal routing in VANET remains a challenging problem. This reason is that efficient route is usually has long link distance while the stable route has short link distance. The present paper addresses this problem by considering hop count and link duration into the routing decision. Geographic routing is promising strategy in VANETs[16]. Geographic routing has outperformed other routing protocols in VANETs because the route information from source to destination need not be stored; such feature is suitable for the high mobility and dynamic nature of VANETs [17, 18]. Therefore, a novel geographic-based routing for finding an optimal route is considered in this study. We propose an efficient and stable routing algorithm based on user mobility and node density (ESRA-MD) in the urban vehicular network. The proposed algorithm involves two phases, namely, the optimal forwarding and neighbor discovery phases. The major contributions of the study are as follows.

1-Two metrics are defined to find the next best candidate node. The intersection selection (*IS*) metric is computed to select the closest intersection near the destination. The weighted distance and connectivity (*WDC*) metric is computed between source and neighbor nodes in one-hop by distance, relative velocity and node density.

2- A next node self-selection method is designed for reducing the communication overhead in congested networks. The method leverages the RTS/CTS exchange to replace the sender selection of the next candidate node.

3- The performance of the proposed ESRA-MD is evaluated by its comparison with adaptive routing protocol based on QoS and vehicular density(ARP-QD)[19],which can also find an optimal and reliable route for data delivery in urban VANETs. However, ESRA-MD offers an improved intersection and next node selection mechanism. The simulation shows that ESRA-MD obtains higher delivery ratio, lower delivery delay, and lower communication overhead than those of ARP-QD.

The rest of the paper is organized as follows. Section 2 presents an overview of the relevant VANET routing protocols. Section 3 introduces the functional details of the proposed routing algorithm. Section 4 analyzes the simulation-based performance evaluation. Finally, Section 5 elaborates the conclusions of the study.

## Related Work

MANETs and VANETs are similar in terms of their ad hoc nature, low bandwidth, and unpredictable communication links. Thus, many studies have modified the routing protocols of MANETs for adopting into VANETs. VANETs are considered a unique type of MANETs and exhibit different features, such as dynamic topological changes, frequent network disconnections, tight delay bounds, no energy limitation issues, geographic-based communication capability, and specific mobility modelling following a road topology [20, 21]. The stability and efficiency of the route are the key design metrics of VANET routing protocols. However, most works focus on either of the two. Some of the existing studies that consider both properties are reviewed below.

In [13], the greedy perimeter stateless routing (GPSR) protocol has been used. This protocol makes next node decisions based on router position and a packet destination. The nearest node to the destination is selected as the next hop, which is within the communication range. However, the high mobility, topology change, and radio obstacles of VANETs increase link loss probability. Hence, significant packet losses and high delays are incurred. In [22], the geographic source routing (GSR) protocol has been used. The calculation of the shortest routing path from source to destination is conducted using map and position data. The junction-to-junction method involves the detection of only the nearest active node without succeeding node while forwarding packets that require GPS. In sparse networks, GSR fails because of its characteristic of maintaining end-to-end connectivity. The rejection of packets occurs because of the occurrence of local maximum by a road segment, which stops the driver from moving ahead to the next available access point. In [14], the greedy perimeter coordinator routing (GPCR) protocol has been used. In this protocol, the nodes at the intersection of the streets have full authority to take routing decisions. The greedy forwarding technique is employed to route packets located between the intersections of the streets. In [23], the greedy traffic-aware routing (GyTAR) protocol has been proposed. This protocol uses the information of the geographical intersections of urban scenarios to determine optimal routing paths. A position-based clustering multi-hop routing algorithm (Clustering) is proposed in [24]. The algorithm uses a neural network method to build the clustering head, which can intelligently distinguish the VANET topology information and partition the network into self-organizing clusters. In summary, these considerable researches focus on achieving the shortest routing path but fail to address other QoS parameters of various applications. Some applications require establishing a stable route to achieve high packet delivery ratio. Meanwhile, the link between the active node and the farthest neighbor node is highly susceptible, thereby resulting in small link duration compared with that of other links. Therefore, the above mentioned protocols are unsuitable for high delivery ratio applications.

In [15], the receive on most stable group-path (ROMSGP) protocol has been proposed. In this protocol, link expiration time is used to measure route stability. ROMSGP is limited by broadcasting only certain packets while losing other packets. ROMSGP offers low packet delivery ratio because the vehicles of similar direction are grouped on the basis of local segment instead of considering the entire route. Delivery delays for ROMSGP are also high because the route with the most hop counts is considered stable. In [25] and [26], the path stability (PASTA) protocol and connectivity aware routing (CAR) protocol have been designed, respectively. The search routes of these protocols exhibit low probability of disconnecting from the network and eliminate carry-and-forward delays. Links with short distance usually exhibit good connectivity; hence, the routes selected by these protocols have many hops, thereby resulting in high delivery delays. In [27], the intersection-based geographical routing protocol (IGRP) has been proposed. This protocol selects the route with high connectivity probability and considers other QoS constraints. In [28], a stable routing protocol (RVD) has been presented for urban VANETs. This protocol works on the real-time information related to traffic density. Every vehicle calculates the road traffic density on which it is present in the beaconing messages received from the vehicles on the opposite lane and through the table containing road information. By considering this obtained road vehicle density information as a routing metric, a reliable routing route is selected by every vehicle. In[29], an epidemic routing protocol (PMIA-EB-ASR) has been proposed for information dissemination in VANETs. An adaptive probabilistic infection and an adaptive limited time forwarding mechanisms are used to ensure a trade-off between the accessibility and efficiency of message dissemination. Moreover, given the self-adaptability and robustness of the cellular gene regulatory networks, the attractor selection mechanism is used. PMIA-EB-ASR drives an informed vehicle to infect a part of its neighbors to be the next forwarding nodes instead of infecting all its neighbors. This protocol also intelligently limits the infection behavior, that is, messages are propagated by the newly infected forwarders. All the above-mentioned works have focused on selecting routes with reliable link connectivity while ignoring the position information of vehicles. This approach causes selection of routes with several loops, thereby resulting in high packet delivery delays. Hence, these protocols are impractical for delay sensitive applications.

The optimized link state routing (OLSR) protocol has been proposed in[30]for determining optimal routes. OLSR uses only the link state to collect information about its neighbors and selects route, which involves the low number of hops as the best route. The design of OLSR considers network topology and includes the exchange of some topology control packets. In [31], an improvement to GPSR has been made by using the speed and position of the vehicle in routing decisions. Speed fluctuations are used to predict vehicle velocities. The aforementioned routing protocols have been designed considering link state and number of hops only and fail to exhibit adaptive behavior for achieving route efficiency or stability required to satisfy the QoS requirements of different applications.

Adaptive routing protocol based on QoS and vehicular density (ARP-QD) has been recently presented in[19], This protocol achieves reliable routes and high packet delivery rates for various urban vehicular networks. ARP-QD also includes a mechanism to lower communication overheads by obtaining the information of the neighbors adaptively from local traffic density. The protocol also mends the broken routes promptly. The use of global distance and speed in optimal forwarding does not cover the QoS parameters of the entire routing path and causes network congestion that affects the packet delivery.

This paper proposes the ESRA-MD algorithm for addressing the limitation of ARP-QD [19]. The novelty of this work lies in its unique design based on distance, relative velocity, and node density in selecting the next best candidate node. In the proposed routing mechanism, the selection of intersection and next node is improved by balancing the route efficiency and route stability through considering hop count and link duration with different QoS requirements in urban VANET environments.

Table 1 presents the differences among protocols in terms of route type, QoS requirement, mobility, and density. Notably, ESRA-MD considers all the factors similar to ARP-QD. Section 4.1 explains the other differences between the two methods.

**Table 1:** Comparison of factors for routing decision making of different routing protocols.

Protocol	Efficient route	Stable route	QoS	Mobility	Density
GPSR [13]	✓	✘	✘	✘	✘
GSR [22]	✓	✘	✓	✘	✘
GPCR [14]	✓	✘	✘	✘	✘
GyTAR [23]	✓	✘	✓	✘	✓
Clustering [24]	✓	✘	✘	✘	✘
ROMSGP [15]	✘	✓	✘	✓	✘
PASTA [25]	✘	✓	✓	✘	✘
CAR [26]	✘	✓	✓	✘	✘
IGRP [27]	✘	✓	✓	✘	✓
RVD [28]	✘	✓	✓	✘	✓
PMIA-EB-ASR [29]	✘	✓	✓	✓	✓
OLSR [30]	✓	✓	✓	✘	✘
SWF [31]	✓	✓	✓	✓	✘
ARP-QD [19]	✓	✓	✓	✓	✓
Proposed (ESRA-MD)	✓	✓	✓	✓	✓

QoS, quality of service; GPSR, greedy perimeter stateless routing; GSR, geographic source routing; GPCR, greedy perimeter coordinator routing; GyTAR, greedy traffic-aware routing; Clustering, position-based clustering multi-hop routing; ROMSGP, receive on most stable group-path; PASTA, path stability; CAR, connectivity aware routing; IGRP, intersection-based geographical routing; RVD, stable routing vehicular density; PMIA-EB-ASR, epidemic routing; OLSR, optimized link state routing; SWF, speed wave forecasted routing; ARP-QD, adaptive routing protocol based on QoS and vehicular density; ESRA-MD, efficient and stable routing based on user mobility and node density.

## Efficient and Stable Routing Algorithm based on User Mobility and Node Density (ESRA-MD)

**Fig 1:**
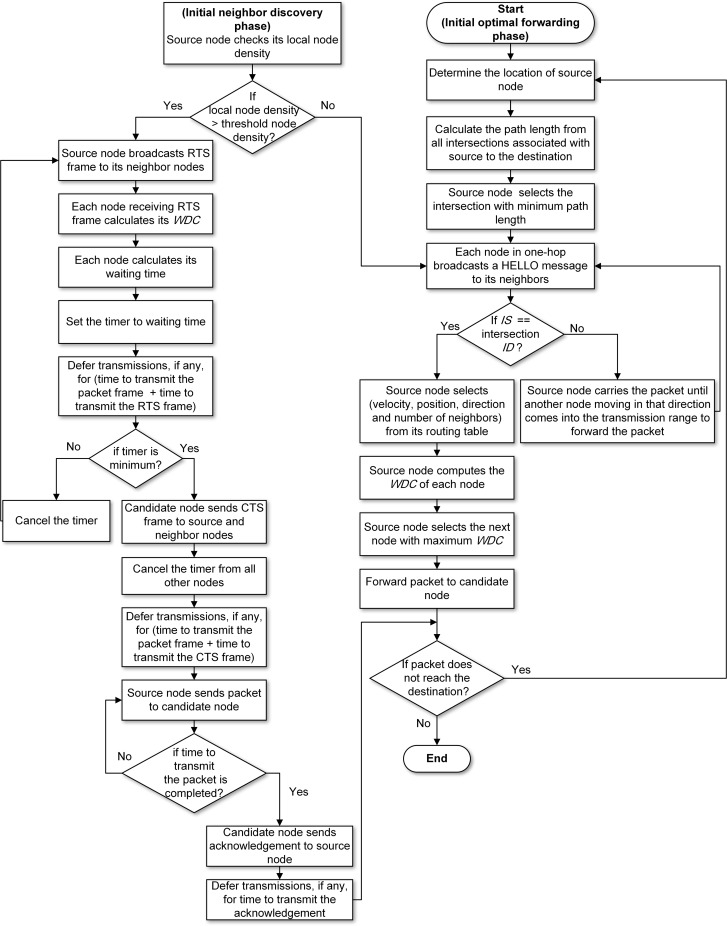
Flowchart of ESRA-MD.

We aim to find an optimal route from source to destination on a hop-by-hop basis. As shown in Fig 1, the ESRA-MD algorithm consists of the optimal forwarding and neighbor discovery phases. The optimal forwarding phase balances the route efficiency and route stability based on the QoS requirements. The neighbor discovery phase eliminates the communication overhead associated with frequent HELLO messages in cases of network congestion.

### Optimal Forwarding Phase

In the optimal forwarding phase, each source routes packets to a destination on a hop-by-hop basis based on local information obtained from its one-hop neighbors. The selection of the next node is based on two metrics. One is intersection selection (*IS*), which is computed for selecting the closest intersection near the destination. The other is weighted distance and connectivity (*WDC*), which is computed between the source and neighbor nodes in one-hop by distance, relative velocity, and number of neighbors. Algorithm 1 illustrates the optimal forwarding phase.

#### Metric Design Intersection Selection (*IS*)

**Fig 2:**
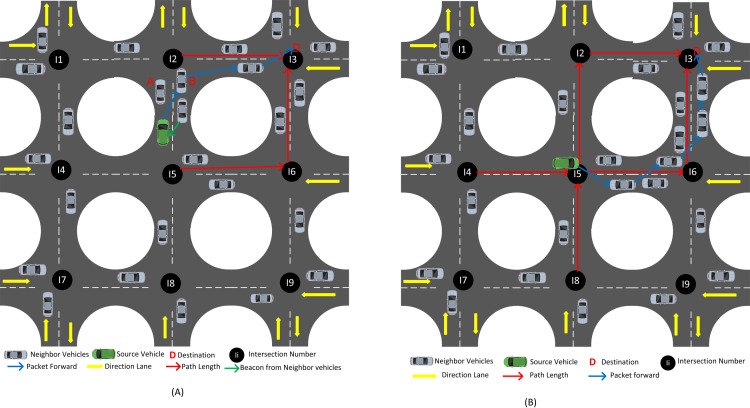
System model. (A) Case 1 source is located in one of the segments. (B)Case 2 source is located in the intersection.

A vehicular network in an urban environment is considered in the system model. This network consists of intersections and segments among intersections. Two lanes are present in every segment wherein vehicles are moving in opposite direction (Fig 2A and 2B). The intersection is represented by the circle with the intersection *"ID"*, vehicle direction in the lane is represented by the arrow, and data center or destination positioned in one of the intersections is represented by the notation *"D"*. We assume that each vehicle can communicate wirelessly and possesses a navigation system according to [32]. Each vehicle consists of a digital map and knows the positions of adjacent intersections. Furthermore, periodic status messages containing information related to the velocity, direction, and position of neighboring vehicle are exchanged periodically and stored in neighbor tables. Without loss of generality, we further assume that all vehicles have the same transmission range, but the offered capacity is subjected to the link level conditions. The source node is assumed to be equipped with location services in acquiring the destination position as and when required [20, 33].

A source needs to select an intersection first and then forwards the packet to the node moving toward the selected intersection. For this purpose, we design an expression that helps in selecting an optimal route as follows:

$$IS=min\left\{PL_{ij}\right\}\;\ldots \ldots \ldots$$ (1)

where *i* is the source node, *j* is the number of intersections, and *PL* is the path length. Packets can be forwarded in two different cases based on the position of the source node. In the first case (Fig 2A), the green vehicle is the source located on segment *I2-I5*, and *D* is the destination located at *I3*. If the source has a packet to send, then it calculates the path length from all intersections associated with it to the destination and selects the intersection with minimum path length. When *PL2D*<*PL5D*, the packet reaching from intersection *I2*-*I3* to *D* is faster than the packet forwarded from *I5*-*I6*-*I3* to *D*. The neighboring gray vehicle comes from the predecessor intersection*I2*and is moving in the opposite direction from the green vehicle. The gray vehicle is sending a beacon to the green vehicle, which contains the *ID* of *I2*. The green vehicle is heading towards *I2*, and identifies *I2* as successor intersection. Then, the source vehicle forwards packet to the vehicle moving in the direction of the intersection with minimal path length to the destination. As illustrated in Fig 2A, vehicles A and B are moving toward the direction of the selected intersection. However, only B will receive the packet from the green vehicle. The reason is that the source vehicle has selected B as the next candidate vehicle depending on the metric of *WDC* shown in Section 3.1.2. The process continues to work until the packetreaches the destination, thereby ensuring rapid progress and less traffic in the network.

In the second case (Fig 2B), the green vehicle approaches intersection *I5*. The vehicle receives a beacon from the neighbor vehicles from four different directions and obtains four intersection *IDs*. The source selects the minimum path length from the intersection to the destination as the next intersection and forwards the packet to the vehicles moving towards that intersection. Therefore, among path lengths*PL4D*, *PL8D*, *PL2D*, and *PL6D*, *PL2D* and *PL6D* are the lowest and have similar lengths. Thus, the packet reaching from intersections *I2* and *I6* to *D* are faster than the packet forwarded from *I4*-*I5*-*I2-I3* to *D* and *I8*-*I5*-*I6-I3* to *D*. Hence, we obtain two paths that have the same path length as follows: *I5-I2-I3* to *D* and *I5-I6-I3* to *D*. In this case, the source can select the path with high number of vehicles for ensuring network connectivity. As illustrated in Fig 2B, the source vehicle selects*PL6* as the path length and selects the vehicle moving between its predecessor and the selected intersection depending on the metric of *WDC* shown in section 3.1.2.

#### Metric Design Weighted Distance and Connectivity (*WDC*)

**Fig 3:**
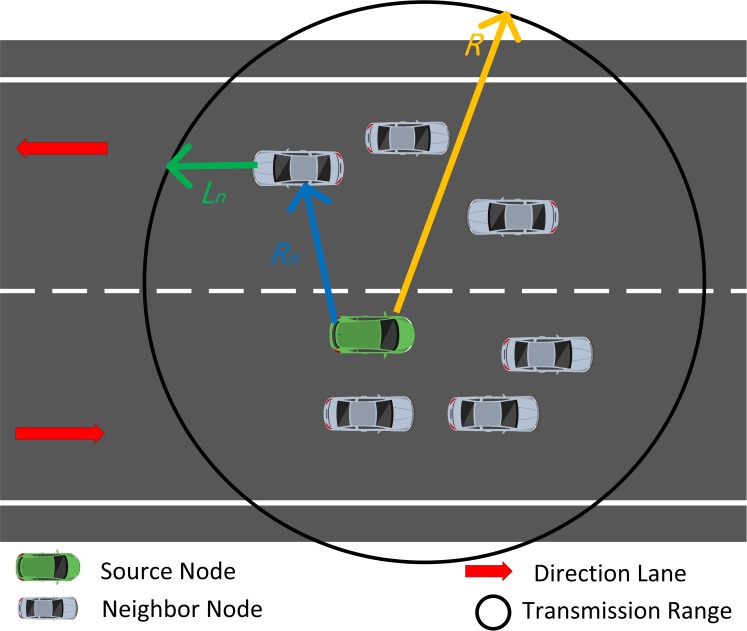
Road segment. R, vehicular transmission range; *Rn*, largest distance of the neighbor that is closest to the destination; *Ln*, distance that the neighbor node moves out from the transmission range of the sender.

We aim to design a routing algorithm that provides route efficiency and improves route stability. Route efficiency means less delay in reaching a destination and can be achieved by forwarding on a route with few hops. Route stability refers to the long link connectivity and can be achieved by considering neighbor density and node mobility in the routing process. Explicitly, we design a novel metric called weighted distance and connectivity (*WDC*), which determines the next best node. As shown in Fig 3, the source node calculates the maximum distance toward the neighbor node, which is the closest to the destination based on the information of position obtained from each node. We adapt Eqs (2) and (3) from [19].

$$R=R_{n}+L_{n}\;\ldots \ldots \ldots$$ (2)

since *PL*_*n*_ < *PL*_*s*_

$$R_{n}=PL_{s}-PL_{n}>0\;\ldots \ldots \ldots$$ (3)

where *n* is the neighbor node, *R* is the vehicular transmission range, *R*_*n*_ is the largest distance of the neighbor that is closest to the destination, *L*_*n*_ is the distance that the neighbor node moves out from the transmission range of the sender, *PL*_*n*_ is the path length between the neighbor node and the destination, and *PL*_*s*_ is the path length between the sender and the destination.

**Fig 4:**
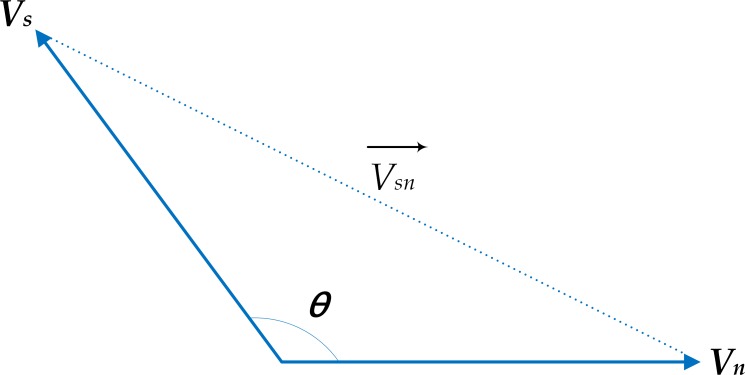
Relative velocities between two nodes moving at an angle *θ*. *v*_*n*_, velocity of the neighbor node; *v*_*s*_, velocity of the current sender; ${\vec{V}}_{SN}$, relative velocity between the source and the neighbor node.

**Fig 5:**
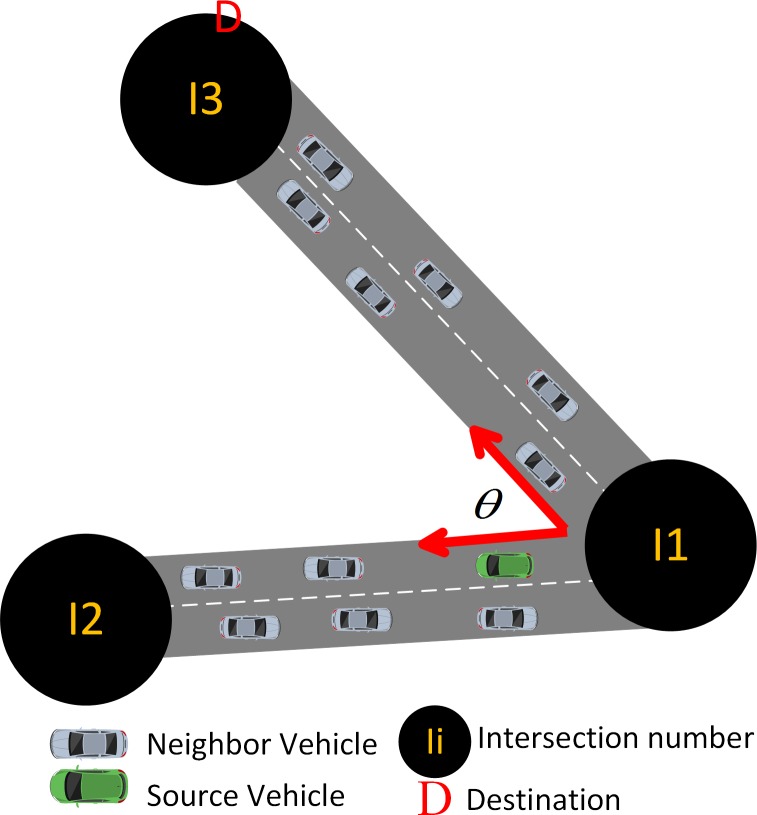
Intersections.

To estimate link stability, the relative velocity between source and candidate neighbor node and the number of neighbors connected to candidate node are calculated. Relative velocity depends on the direction of nodes. In analyzing the relative velocity parameter, Fig 4 considers a neighbor node *n* and a source node that move in different directions. Consequently, an angle *θ* exists at the intersection. By using the cosine law, the relative velocity between the source and the neighbor node is given by (4):

$${\vec{V}}_{SN}=\left\{ \begin{array}{c} \sqrt{v_{s}^{2}-2v_{s}v_{n}\;\cos\theta +v_{n}^{2}},\;\theta \neq 0\;or\;180\\ v_{s}-v_{n},\;\theta =0\\ \;v_{s}+v_{n},\;\theta =180 \end{array}\right.\ldots \ldots \ldots$$ (4)

where *v*_*n*_ is the velocity of the neighbor node, *v*_*s*_ is the velocity of the current sender, and ${\vec{V}}_{SN}$ is the relative velocity between the source and the neighbor node. According to (4), the following three movement cases can be obtained depending on *θ*:

1. *θ* ≠ 0 or 180, in which source and candidate neighbor are in different segments and different directions (Fig 5). The figure considers the green vehicle as the source moving toward intersection *I2* from intersection *I1*, while the destination is placed at the next segment. Hence, the source makes an angle *θ* with the candidate vehicle, which is moving in a segment other than from that of the source.
2. *θ* = 0, in which source and candidate neighbor are moving in the same direction in the same segment.
3. *θ* = 180, in which source and candidate neighbor are moving in opposite direction in the same segment.

Accordingly, we select the node with less difference in relative velocity either in the same direction or opposite direction. This choice helps in maintaining the duration of link connectivity for a long period. A number of neighbors (*N*_*neigh*_) help maintain the link stability and fast link recovery. High number of neighbors means high probability of obtaining a subsequent route to the destination if a link fails. Hence, to balance route efficiency and route stability, we select the candidate neighbor node withthe next hop of large distance, less relative velocity, and high number of neighbor nodes. We define a metric *WDC* as follows:

$$WDC=\alpha R_{n}+\beta (\frac{N_{neigh}}{{\vec{V}}_{SN}})\;\ldots \ldots \ldots$$ (5)

where we assume that large value of *R*_*n*_ means less hop count, while low value of ${\vec{V}}_{SN}$ and high value of *N*_*neigh*_ indicate improved connectivity and stability. Therefore, a source node prefers to choose a candidate node *n* with a maximum value of *WDC*, where *α*, *β* are constant weights. As a result, the system requirements, such as route efficiency and route stability, are specified. Once the source obtains information about velocity and position from its neighbors, it computes the *WDC* of all the neighboring nodes in its neighbor list. If multiple neighbor nodes with the same large *WDC* exist, then the source node will randomly pick one as the next node. When a link failure is encountered in VANETs, the concept of carry and forward is used by the source node to start searching for routes locally [34]. The one-hop neighbors are explored for finding backup links in case of failed links. If no hop neighbor exists on the required direction, then the source waits for a specific period until another in-range neighbor is found in the chosen direction. Hence, the routing performed by this carry and forward technique is loop free without any endless forwarding loops because transmission can be made on already traveled hops. The algorithm considers two QoS requirements, namely, delivery delay and delivery ratio. Delivery delay is significant for applications, such as video conferencing and video on demand. Thus, we require route with less hop count. Meanwhile,message delivery ratio is important for applications, such as file transfer. We use factors *α* and *β* to represent diverse QoS requirements for different applications. In this part, *α* represents the priority weight for hop count, while *β* represents the priority weight for link connectivity under the condition of *α* + *β* = 1. We can adjust the weights to satisfy various QoS requirements for different applications.

**Algorithm 1**. **Optimal forwarding phase.**

**Input**: The information of sender and destination nodes

**Output**: The next candidate node

**Notation**:

*Sn*: Source node

*PL*: Path length from all intersections associated with source to the destination

*x*_*s*_: Location of source intersection

*x*_*d*_: Location of destination intersection

*IS*: Intersection selection

*N*_*neigh*_: Number of neighbors

        1. **Begin Algorithm** **1**

        2. Determine the location of *Sn*, which has a packet to send;

        3. Calculate PL = $\sqrt{({x_{sx}-x_{dx})}^{2}+{{(x}_{sy}-x_{dy})}^{2}}$;

        4. *Sn* selects the intersection with minimum *PL*;

        5. Each node in one-hop broadcasts a HELLO message to its neighbors;

        6.       **If** intersection *ID* equals to *IS*, **then**

        7.           *Sn* selects velocity, position, direction, and *N*_*neigh*_ from its routing table for those neighbors;

        8.            *Sn* computes the *WDC* of each node;

        9.            *Sn* selects the next candidate node with maximum *WDC*;

        10.            Forward packet to candidate node;

        11.       **Else**

        12.            *Sn* carries the packet until another node moving in that direction comes into the transmission range to forward the packet;

        13.            Go to 5;

        14.        **End If**

        15.                **If** packet does not reach the destination, **then**

        16.                    Go to 2;

        17.                **End If**

        18. **End Algorithm 1**

### Neighbor Discovery Phase

**Fig 6:**
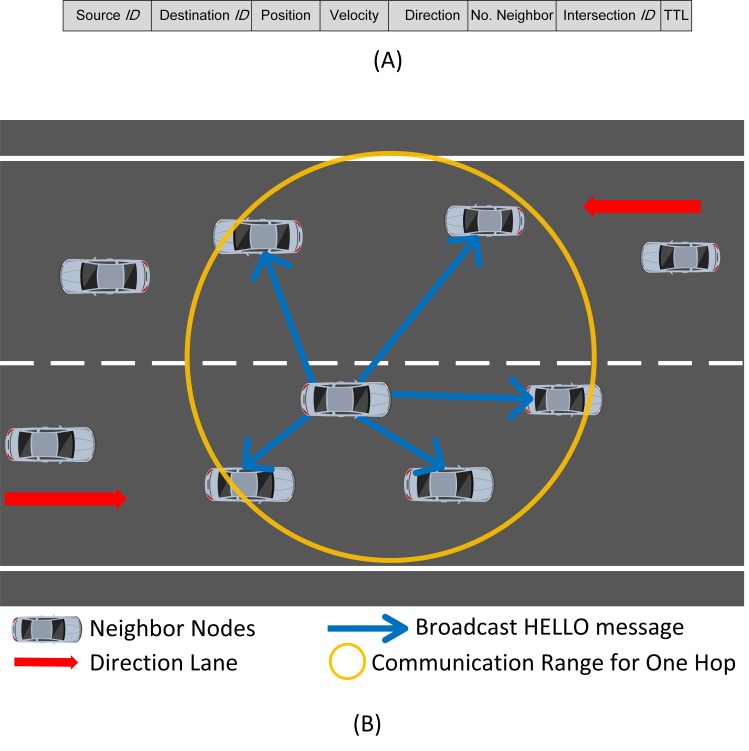
HELLO message. (A) HELLO message frame. (B) HELLO messages to exchange information.

In the neighbor discovery phase, each node transmits a HELLO message (Fig 6A) to exchange information with its neighbors at regular intervals. This real-time information is stored in the neighbor tables maintained by every node. Node density significantly affects the network performance, and high node density incurs serious congestions during the update process of neighbor information. Thus, periodic HELLO messages add to routing overhead, affect data transmission, and causes network congestion. A distributed hop selection method proposed in [35]has been used as a solution to the mentioned problem in this phase. This method increases packet delivery ratio in congested networks while reducing communication overhead encountered in the selection of next node. The neighbor discovery phase locates neighbors to upgrade the neighbor table depending on node density. Therefore, two metrics are defined as follows: 1) local node density (*d*_*l*_), which defines the neighbors of node *i* within its transmission range; and 2) threshold node density *(d*_*th*_), which is used to evaluate *d*_*l*_. The initiation of the neighbor discovery phase is marked by the source node when it evaluates its *d*_*l*_ for determining the next best node. If *d*_*th*_is higher than *d*_*l*_, then fewer communication overheads will occur during the neighbor discovery phase. Every node broadcasts a beaconing HELLO message to its neighbors, as shown in Fig 6B. In this way,the neighbor table is updated by every node. Considering the information gathered from neighboring nodes, the packet is forwarded by the source node to the node that is selected as the next best node depending on the *WDC* value by (5). The entire process is repeated until the packet reaches the destination.

**Fig 7:**
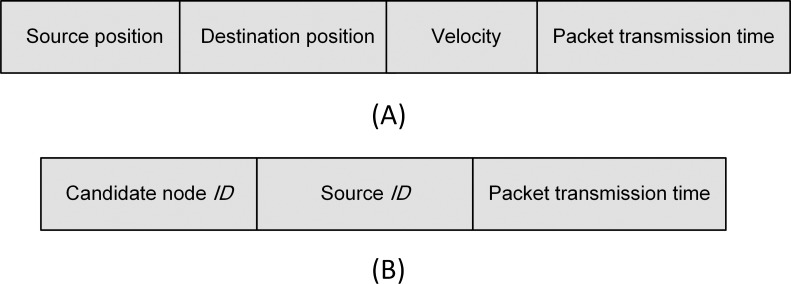
RTS/CTS. (A) RTS frame. (B) CTS frame.

Conversely, if *d*_*th*_is lower than *d*_*l*_, then high communication overheads are encountered during the neighbor discovery phase. Hence, a solution has been proposed in the form of ESRA-MD in which the selection of next node by the source node is replaced by leveraging RTS/CTS messages. Accordingly, communication overhead caused by frequent HELLO messages is eliminated in the cases of network congestion. The proposed method uses the concept of receiver-based relay selection technique in its design. The currently used techniques of this method use implementation distance between next node and destination node as the measure for calculating waiting time. However, the proposed method uses two additional metrics, namely, relative velocity and number of neighbors. Furthermore, the next node self-selection method in the proposed algorithm performs piggybacking of data on the IEEE 802.11 RTS/CTS frames in reducing communication overhead. Thus, Fig 7A shows that an RTS frame is broadcasted by the source node to its neighbor nodes. After receiving the RTS frame, every node calculates its own *WDC* value by (5) and a waiting time. A CTS frame is then sent back to the source node, as depicted in Fig 7B. The waiting time determines whether the node can serve well as a forwarding candidate node. Therefore, if the waiting time is small, then the node becomes the best candidate node to forward the packet. The following formula is used to calculate the waiting time:

$$Tw=\frac{T}{WDC}\ldots \ldots \ldots$$ (6)

where *T* is a parameter that indicates time and is set by the vehicular network. The parameter regulates the relation between the waiting time and *WDC* value of receiver. The source forwards the packet to the next best candidate node after receiving the CTS. The next node sends an acknowledgment of the packet receipt. Algorithm 2 illustrates the neighbor discovery phase.

**Fig 8:**
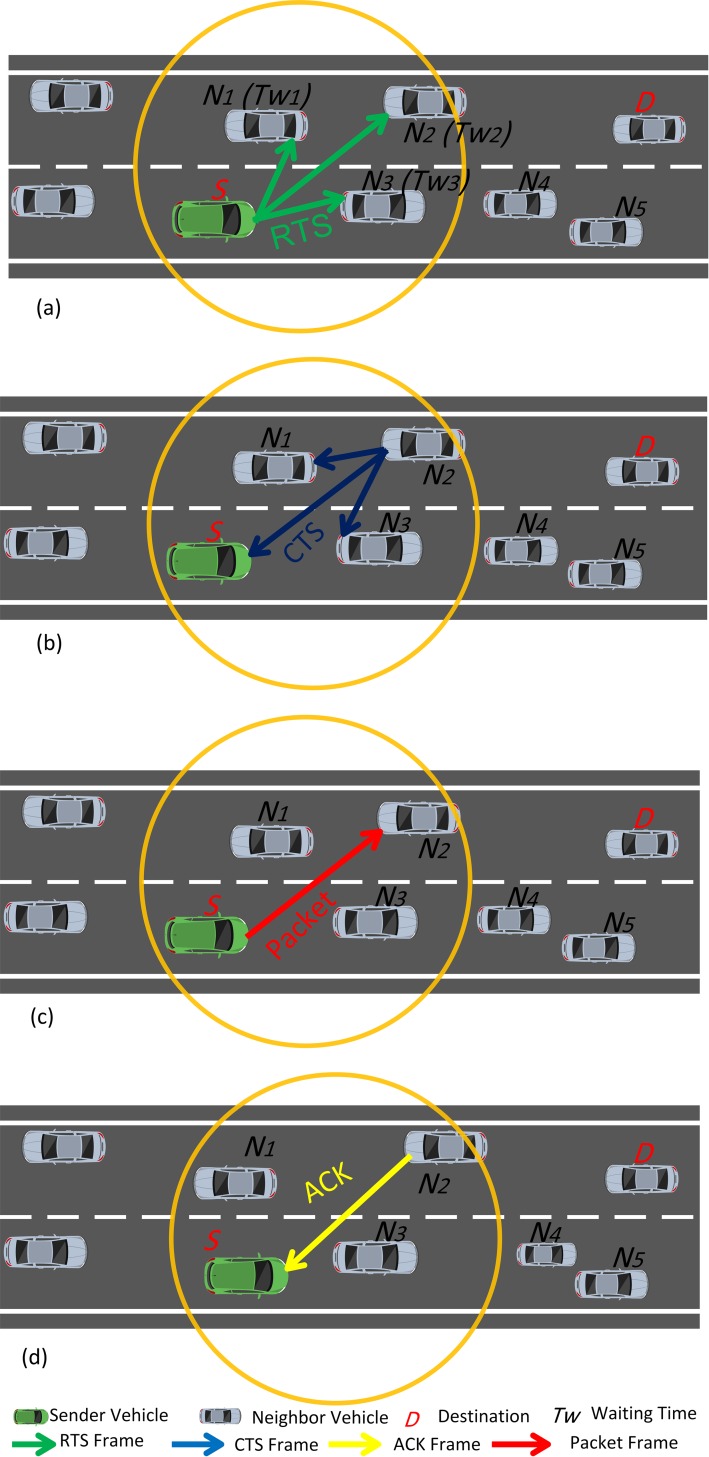
Next node self-selection method. S, source; D, destination; N, neighbor; RTS, request to send; CTS, clear to send; ACK, acknowledgment.

The next node self-selection method is illustrated in Fig 8. Source *S* searches for the next node to forward the packet to destination *D*. The source specifies its own position and that of destination node and its own velocity and transmission time of packet. The source also broadcasts an RTS frame. This RTS frame is overheard by nodes *N1*, *N2*, and *N3*, which compute their waiting time and hold their timers in waiting for mode before they reply to source *S* by sending CTS frame. We assume that the shortest waiting time (*Tw2*) is that of node *N2* and that this node is the first one to send CTS frame. This CTS frame is overheard by *N1* and *N3*, and they then cancel their timers. Moreover, the neighbors of *N2*will know that a transmission is ongoing and that they must not send any frame to *N2* until it completes the transmission. On receiving CTS frame from *N2*, the packet frame is sent to *N2* from *S*. This transmission is overheard by the neighbors of *S*, and they learn that they must not send any frame to *S* until *S* receives an acknowledgment from *N2*. In this way, the next node is efficiently chosen without transmitting any HELLO message overhead.

**Algorithm 2**. **Neighbor discovery phase.**

**Input:** The local node density(*d*_*l*_) of node *i*, the threshold node density (*d*_*th*_)

**Output:** The neighbor table of node *i*

**Notation**:

*Sn*: Source node

*Twaiting*: Waiting time

*Snposition*: Source node position

*Dnposition*: Destination node position

*V*: Velocity of source node

*Tpacket*: Time to transmit the packet frame

*TRTS*: Time to transmit the RTS frame

*SnID*: ID of the sender that searches for the next node

*NID*: ID of the next candidate node

*TCTS*: Time to transmit the CTS frame

*TACK*: Time to transmit the ACK frame

        1. **Begin Algorithm** **2**

        2. *Sn* checks its *d*_*l*_;

        3.      **If** *d*_*l*_**<***d*_*th*_, **then**

        4.          Run the command line 5 until the line 18 of Algorithm 1;

        5.     **Else**

        6.          *Sn*broadcasts RTS (*Snposition*, *Dnposition*, *V*,*Tpacket*) to its neighbor nodes;

        7.           Each node receiving RTS frame calculates its *WDC*;

        8.           Each node calculates its *Twaiting*;

        9.           Set the timer to *Twaiting*;

        10.           Defer transmissions, if any, for *Tpacket* + *TRTS*;

        11.               **If** timer is minimum, **then**

        12.                   Candidate node sends CTS (*NID*, *SnID*, *Tpacket*) to *Sn* and all neighbor nodes before the timeout;

        13.                   Cancel the timer from all other nodes;

        14.                   Defer transmissions, if any, for *Tpacket + TCTS*;

        15.                   *Sn* sends packet to candidate node;

        16.   **If** *Tpacket* is completed, **then**

        17.       Candidate node sends *ACK* to *Sn*;

        18.     Defer transmissions, if any, for *TACK*;

        19.                               Run the command line 15 until the line 18 of Algorithm 1;

        20. **Else**

        21.     Go to 15;

        22.             **End If**

        23.              **Else**

        24.                     Cancel the timer;

        25.                     Go to 6;

        26.       **End If**

        27.     **End If**

        28. **End Algorithm** **2**

## Performance Evaluation and Result Analysis

We evaluate the performance of the proposed ESRA-MD by its comparison with ARP-QD using a MATLAB simulator. In this section, we present the differences between ESRA-MD and ARP-QD first and then the performance analysis.

### Differences between ESRA-MD and ARP-QD

Similar to ESRA-MD, the ARP-QD [19] is capable of finding an optimal and reliable route for end-to-end data delivery within urban VANET environments according to diverse QoS requirements. Table 2 shows that the two methods have the same objective, but they differ in certain aspects.

**Table 2:** Differences between ESRA-MD and ARP-QD.

ESRA-MD	ARP-QD
• In all source cases, distance, relative velocity, and number of neighbors used for selecting the next best candidate node. • Intersection selection is always performed first prior to finding the next best node. • The next candidate intersection is selected based on path length. • A beacon packet that consists of a HELLO message only is broadcasted. • Distance, relative velocity, and number of neighbors used for selecting the waiting time in the next node self-selection method.	• In all source cases, distance and velocity used for selecting the next best candidate node. • Intersection selection is only conduced when source approaches an intersection. • The next candidate intersection is selected based on path length, the angle between source and next candidate node, and connectivity. • A beacon packet that consists of a connectivity probe request and a connectivity probe reply is broadcasted. • Distance and velocity used for selecting the waiting time in the next node self-selection method.

ESRA-MD, efficient and stable routing based on user mobility and node density; ARP-QD, adaptive routing protocol based on QoS and vehicular density.

### Simulation Results

In the performance evaluation, we first focus on delivery ratio and delivery delay in observing the effect of weighted factors α and β. We then focus on communication overhead. Table 3 summarizes the key parameters in the simulation.

**Table 3:** Simulation parameters.

Parameter	Number of Value
Intersections	9
Segments	12
lanes	2 bidirectional
Number of nodes	100, 200, 300, 400, 500
Velocity	10–30 m/s
Simulation duration	600 s
Simulation area	5×5 km^2^
Local node density	20 veh/lane/km
Threshold node density	40 veh/lane/km
Bit rate	6 Mbps
Wireless communication range	500m
Mac protocol	IEEE 802.11p
Data packet size	512 Bytes
RTS frame size	20 Bytes
CTS frame size	14 Bytes
ACK frame size	14 Bytes
Beacon packet size (HELLO message)	64 Bytes
CBR rate	20 bit/s
Carrier frequency	5.9 GHZ
Transmission rate	1, 2, 3, 4, 5 packet/s
Routing protocol	ESRA-MD, ARP-QD
Antenna type	Omni-directional
Path loss	Free space propagation loss (FSPL)
Mobility model	Car-followingmodel (CFM)

RTS, request to send; CTS, Clear to Send; ACK, acknowledgment; CBR, connection bit rate.

#### Delivery ratio

**Fig 9:**
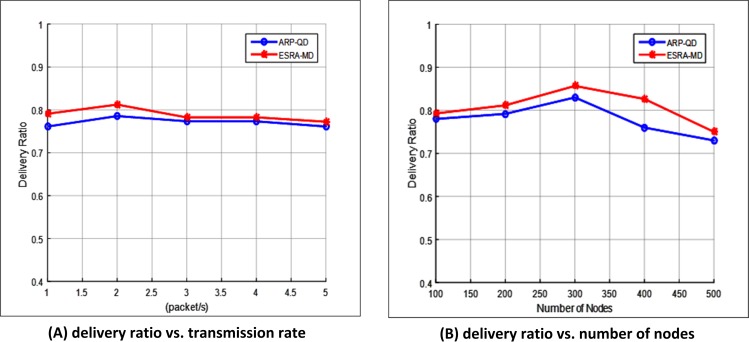
Delivery ratio. (A) Delivery ratio vs. transmission rate. (B) Delivery ratio vs. number of nodes.

Delivery ratio measures the ratio of total data packets successfully delivered to the destination node to the data packets generated by the source node. The packets are dropped if the TTL hits zero or it fails to deliver the link fails. Fig 9A and 9B illustrate the delivery ratio with varying transmission rates and number of nodes, respectively. The two figures show that the proposed ESRA-MD exhibits a slightly higher delivery ratio than ARP-QD in all cases. The reason is that intersection selection in ESRA-MD is always performed prior to finding the next best node. By contrast, ARP-QD works on the intersection selection only when the source approaches an intersection, thereby leading to high number of dropped packets. Moreover, ESRA-MD uses density in selecting the next best candidate node. Such approach significantly affects the stability of inter-vehicle communication links in VANET. This finding is explained by the highest connection probability provided by the routes selected by ESRA-MD. Therefore, ESRA-MD is effective and less prone to failure when finding a route toward the destination. As a result, high number of packets is needed to reach the destination. Therefore, the delivery ratio in ESRA-MD is higher than that in ARP-QD.

Fig 9A shows that the delivery ratio of ESRA-MD is increased at transmission rate 1 and reaches the highest delivery ratio of 0.81 at transmission rate 2. This result is due to that the local node density in this period is higher than the threshold density. In other words, the next node self-selection method is provided in the neighbor discovery phase. Therefore, the communication overhead reduces in this period, thereby increasing the delivery ratio. At transmission rates between2.5 and 5, the delivery ratio of ESRA-MD decreases until it reaches 0.76. This finding is attributed to that the average throughput of the network is decreased because the local node density in this period is lower than the threshold density. Such condition means that the periodic beacons are provided in the neighbor discovery phase. Therefore, the communication overhead increases in this period, thereby decreasing the delivery ratio. Fig 9A shows that the delivery ratio of ESRA-MD is 3.84% higher than that of ARP-QD with different number of transmission rates. In Fig 9B, the delivery ratio for ESRA-MD increases when the number of nodes is 300and achieves the highest value of 0.85. The reason is that, before reaching 300, the threshold density is less than the local node density. Hence, during the neighbor discovery phase, less communication overhead is encountered because of the next node self-selection method. In turn, the delivery ratio is increased. However, the delivery ratio for ESRA-MD decreases gradually until it reaches 0.75 when the number of nodes is between 350 and 500. This finding is explained by that the threshold density is higher than the local node density. Hence, the communication overhead increases because the periodic beacons are provided in the neighbor discovery phase. Given that the neighbor table needs to be updated for all neighbors, the delivery ratio decreases. Fig 9B shows that the delivery ratio of ESRA-MD is 7.79% higher than that of ARP-QD at different number of nodes.

#### Delivery delay

**Fig 10:**
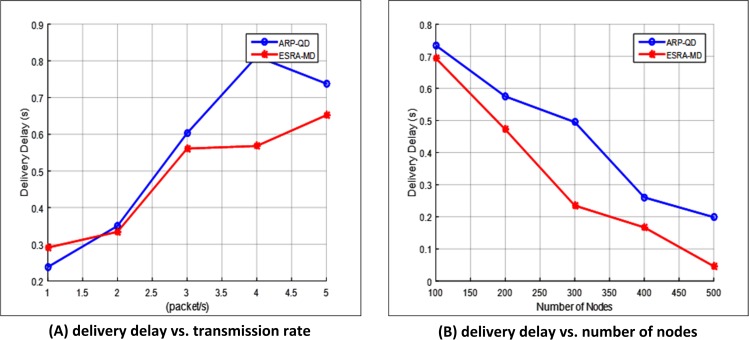
Delivery delay. (A) Delivery delay vs. transmission rate. (B) Delivery delay vs. number of nodes.

Delivery delay is the difference between the time a packet is received at the destination and the time the packet is sent by the source. Fig 10A and 10B illustrate the delivery delay with varying transmission rates and number of nodes, respectively. The figures indicate that ESRA-MD produces lower delays than ARP-QD for both cases. The delay increases because of the increase in the number of hops and a failure in links. ESRA-MD always finds a node near the intersection that is very close to the destination. The algorithm also checks for the next intersection on each node before forwarding the packet. Moreover, ESRA-MD always selects the route with high number of nodes to ensure network connectivity, especially when more than one route has the same path length. The algorithm avoids the packet that will be transmitted away from the destination. On the contrary, ARP-QD works on intersection selection when the source approaches an intersection; thereby leading to high number of dropped packets and delays. Therefore, the delivery delay in ESRA-MD is lower than that in ARP-QD.

Fig 10A shows that the increase in the transmission rate increases delivery delay. The reason is that the source in ESRA-MD cannot find a backup neighbor in case of failure because of the high transmission rate. Hence, the carry forward procedure is prolonged and the delivery delay increases. Moreover, the delivery delay increase slightly to 0.29 and 0.33s when the transmission rate is between 1 and 2 because of the high delivery ratio in this period (Fig 9A). However, the delivery delay increases significantly to 0.45 and 0.65s when the transmission rate is between 2.5 and 5given the low delivery ratio in this period (Fig 9A).Fig 10A shows that the delivery delay of ESRA-MD is 41.3% lesser than that of ARP-QD with different transmission rates. Fig 10B shows that the delivery delay decreases along with the increase in the number of nodes. The delivery delay decreases dramatically to 0.7–0.23s with nodes between 100 and 300 as a result of the high delivery ratio in this period. The next node self-selection method is provided in this period to select the best node candidate. This method depends on the RTS/CTS frame, which chooses the node that has short waiting time. Therefore, the packet reaches to the destination with minimum delay. Moreover, when the number of nodes is between 350 and 500, the delivery delay continuously decreases to 0.2 and 0.05s. ESRA-MD uses maximum distance in *WDC* metric for selecting the next best candidate node. Therefore, the packet reaches to the destination with low delay and few numbers of hops. Fig 10B shows that the delivery delay of ESRA-MD is 43.7% lesser than that of ARP-QD at different number of nodes.

#### Effect of Weighted Factor α

**Fig 11:**
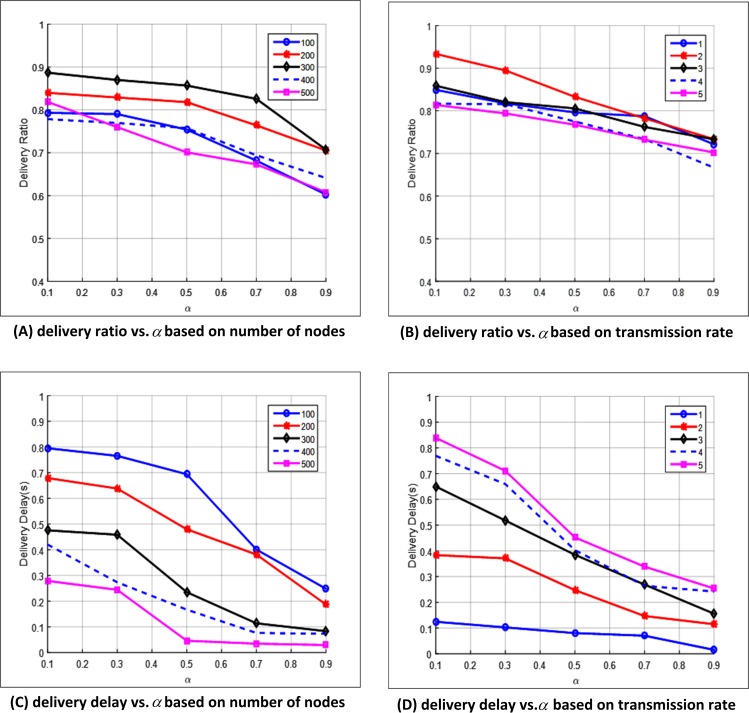
Effect of weighted factor *α*. (A) Delivery ratio vs. *α* based on number of nodes. (B) Delivery ratio vs. *α* based on transmission rate. (C) Delivery delay vs. *α* based on number of nodes. (D) Delivery delay vs. *α* based on transmission rate.

The effect of weights α and β is evaluated for the diverse QoS requirements by varying the simulation results of delivery ratio and delivery delay while increasing the value of α from 0.1 to 0.9. The transmission rate is fixed to 5packet/s in Fig 11A and 11C, and different number of nodes is represented by five curves. In Fig 11B and 11D, the number of nodes is fixed to 300 while transmission rates are denoted by five curves.

Fig 11A and 11B illustrate the variation in delivery ratio with the change inα based on fixed number of nodes and transmission rate, respectively. Fig 11C and 11D illustrate the variation in delivery delay with the change in α based on fixed number of nodes and transmission rate, respectively. The figures show that that the delivery ratio declines while the delivery delay goes down with the increase inα. The reason for this behavior is that the increase inα value results in large link efficiency weighs and small link stability weighs. The link breaks with the increase in α, thereby explaining the low delivery ratio. The improvement of delivery delay is due to the few number of hops with strong requirement of link efficiency. Fig 11B shows a constant delivery ratio with different transmission rates, similar to that observed in Fig 9A. Fig 11D exhibits varying delivery delay with different transmission rates, similar to that observed in Fig 10A.These results show that weighing factor α can cater to the different QoS requirements of various applications.

#### Communication overhead

**Fig 12:**
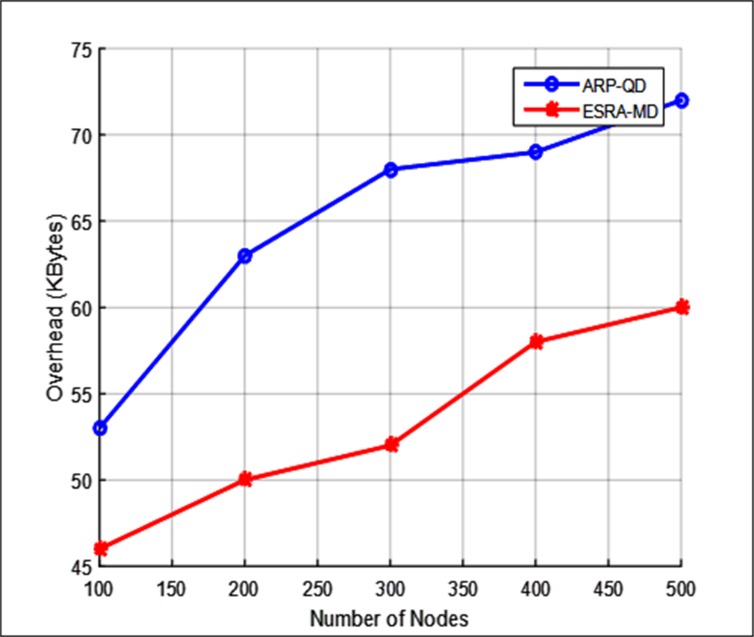
Communication overhead.

Communication overhead is the number of control messages sent by the routing protocols to construct and maintain their routes. Fig 12 illustrates communication overhead withvarying number of nodes. Fig 12 shows that the overhead for ESRA-MD is less than that for ARP-QD in all number of nodes. The reason is that, during a neighbor discovery phase, ESRA-MD only has a periodic HELLO message whereas the ARP-QD has a connectivity probe request and a connectivity probe reply. The ESRA-MD curve shows that the communication overhead increases with the increase in number of nodes. This result is due to that, when the number of nodes is below 300, the local node density becomes higher than the threshold density. Hence, during the neighbor discovery phase, less communication overhead is encountered because of the next node self-selection method. Therefore, the communication overhead is equal to approximately 45 and 53 Kbytes. However, for the number of nodes above 300, the local node density becomes lower than the threshold density. Hence, the communication overhead increases because the periodic beacons are provided in the neighbor discovery phase. Accordingly, the communication overhead increases to approximately 53 and 60 Kbytes. Fig 12 shows that the communication overhead of ESRA-MD is 30.7% lower than that of ARP-QD with different number of nodes.

## Conclusion

The proposed ESRA-MD provides a fast and reliable route for end-to-end data delivery, thereby satisfying diverse QoS requirements in urban VANET environments. The novelty of this work lies in its unique routing algorithm based on distance, relative velocity, and node density. ESRA-MD provides the best way to balance route efficiency and route stability by considering hop count and link duration. Moreover, ESRA-MD provides a solution for reducing the communication overhead associated with the selection of the next node in congested networks based on RTS/CTS frames. The simulated results show that ESRA-MD obtains slightly higher delivery ratio and lower delivery delay than ARP-QD with varying transmission rates and number of nodes. In addition, the communication overhead for ESRA-MD is less than that for ARP-QD regardless of node density.
